# Separating Teeth

**Published:** 1874-07

**Authors:** W. G. A. Bonwell


### ARTICLE VI.

### *Separating Teeth.*

BY W. G. A. BONWELL, D. D. S.

The use of the diamond in the permanent separation of the incisors, superior and inferior, and the hard rubber corundum disks for the bicuspids and molars, will now form the subject of greatest vital importance.

To restore to nature by art that which has been lost, and as in the teeth, the members made useful for a longer period, is a task of which any of us may feel proud. But, to anticipate the tooth of time and make restoration unnecessary is by far a prouder task, and when performed, will elevate the profession to a much higher standard.

What are we doing to day to rid the world of the ravage of decay in the human teeth? Have we done one thing to stay its progress? Are we doing for our patient all that we are conscious is our duty in lessening the great number of fillings, and the greater number of artificial dentures? I say in all good faith, no! no!

Many have, on a small scale, so acted, but the means hitherto at our hands have been inadequate to the end. It is my pleasure to demonstrate to you to-day that we have at our doors the coveted instruments whereby we can say to that insidious sapper, "thus far shalt thou go and no farther."

The old way of separating teeth, by file and chisel, was bad enough to practice when actually demanded, where decay was present, and *filling* necessary; but to use them on the approximal surface of every tooth to anticipate decay, was too barbarcos. But few would permit it. As a fixed practice it could not be done, and when done could not be fully relied upon from the impossibility of separating sufficiently at the cervical walls, and even when done, of placing such a smooth surface thereon as would prevent decay without constant attention.

What have been the steps taken to take the place of the file and chisel? I think I am safe in saying that the first disk ever given to the profession was a steel file of one inch diameter, recommended to those who bought my drill, the first foot-engine in the market, though not the first patented. Upon this Dr. Arthur's shellac and corundum disks came forth, which, if there were nothing more permanent, are nearly all that we could desire.

When hard rubber plates first came out I made of rubber and emory, separating files, instead of steel, whereby I could cut tooth substances or dress up fillings. For want of proper means of mixing rubber and emory I abandoned it. This old idea is now reproduced, and this disk which I show you, has been the result. With these it becomes an easy and inexpensive task to separate every back tooth, and so perfectly that decay can be anticipated on every approximal surface, if done as soon as the permanent teeth have arranged themselves in the arch. Do not wait a day; for delays are dangerous. The pain is comparatively nothing, and the inconvenience to the patient is of no consideration. The hardest teeth can be separated in three minutes. Their great strength, with their thinness and delicacy, are as ten to one to shellac, and no fear need be entertained of doing injury to the mouth when run at 4,000 revolutions per minute. It is the work of but a few moments in removing decay where superficial, or, which is better still, to separate before decay has begun. Every one knows that at least ninety per cent. of the cases coming under their care, every approximal surface, or nearly all, have to be filled by the time they arrive at the age of thirty. The majority sooner.

When separated while the tooth structure is yet sound the division is not wide, and such a solid shoulder or abutment can be left that not only prevents any closer approach, but is never a source of annoyance to the patient as when left to decay and then fill, and in quite every case no solid cervix is left to bear the pressure. By this means we can prevent every approximal surface from further decay, and save immensely in every way to the public.

But the disk will not separate more than the bicusps and molars. What have we for completing the arch? I present to you to day the diamond pointed reamer, with a permanent steel back-action for the specific purpose of separating from the palatal side of the superiors, and lingual side of inferior incisors all their proximal surfaces. The superiors I separate whether decay is present or not. The inferiors never, unless decay is present.

The operation is done entirely from the inside of the mouth, and such a groove is made in the short space of five minutes, as not only removes all predisposition to caries, but the division is not seen from the labial surface other than a mere trifle. The entire beauty of the tooth is preserved without any danger from future decay. The most perfect division is made that is possible for any other point to make but that made by a three or five angled diamond pyramid point. It is the thing for a specfiic purpose, and with its conscientious use, with that of the hard rubber corrundum disk, there need not be an artificial denture from caries alone. If we wait a wider division, one or more fillings are required, and probably refilling in a few years, with all the annoyance of food pressing upon the gums.

My plan is to commence with children at three years of age, or as soon as the temporary are in place, and separate every proximal surface with the disk, front and all, except the inferior incisors. Sometimes use the diamond reamer on front teeth. Principally the disk for children, Do not wait until the first permanent molars appear. It is too late to do so perfectly. Then by filling the grinding surfaces, as well as any other places that could not be cut out, we are sure of their temporary teeth. When the permanent take their place I at once make the disk and diamond reamer do their work.

My experience of twenty years confirms me in the assurance that I am performing a more noble and lasting work than by waiting, and having to restore by filling. No one can deny the facts that every day stare us in the face, that if we would have the greatest number of human teeth preserved we must resort to this wholesale treatment. The experience of the old dentists confirms me that I am right, and not a day passes but I see the wisdom of such a policy. In some cases it seemed almost like sacrilege to cut beautiful teeth, but in scores of cases, where I least suspected decay being present, I have found it deeply seated. By no other means could it have been detected.

I have yet to regret the first case where this simple treatment has been maintained. I repeat, instead of waiting for the first molars to appear, commence by dividing every temporary tooth, except inferior incisors, and you have done the best work, that of anticipation. By all means preserve the deciduous by dividing and filling with even ordinary tin foil when needed. Until these means were at my hands I would not have undertaken such Herculean labor, nor would I have subjected my patients to the pain incident thereto, but with these it is but the work of a moment, and a labor that not only pays the patient but the operator, and endows a clean conscience that preservation is nobler than restoration.—*Proceedings of Harris Dental Association.*

